# Auxin accumulation in cereals after infection by *Fusarium graminearum*: putative biosynthetic pathways and preferences

**DOI:** 10.1007/s44154-025-00267-0

**Published:** 2026-01-29

**Authors:** Huanzhang Shang, Bo Ji, Thérèse Ouellet, Guangwei Li, Boliao Li, Xiulin Chen, Kun Luo

**Affiliations:** 1 Key Laboratory for Applied Ecology of Loess Plateau (Shaanxi Province), College of Life Science, Yan’an University, Yan’an, 716000 Shaanxi China; 2 Ottawa Research and Development Centre, Agriculture and Agri-Food Canada, 960 Carling Ave, Ottawa, ON K1A 0C6 Canada

**Keywords:** Auxin biosynthesis, L-Tryptophan-dependent, Tryptamine metabolism, Phytohormones crosstalks, Scab-resistant, Molecular breeding

## Abstract

Indole-3-acetic acid (IAA) is a major naturally occurring auxin that shows extensive accumulation in cereal plants during the first few days of infection by the phytopathogen *Fusarium graminearum*. Apart from its positive effects on plant growth, empirical studies have suggested that it is a virulence factor that alters the host’s nutritional level and fine-tunes the plant’s immune responses, especially salicylic acid-mediated defenses. Plant and fungus genomic studies have predicted that their genomes carry the required genes for L-tryptophan-dependent IAA biosynthetic pathways. In recent decades, genetic and genomic studies have facilitated the description of L-tryptophan (L-TRP)-dependent IAA biosynthetic pathways in *F. graminearum* and its host plants. The present review illustrates and summarizes the putative and preference molecular networks related to extensive IAA accumulation in wheat heads triggered by infection with *F. graminearum*, based on the available knowledge about the endogenous IAA biosynthetic pathways in *F. graminearum* and wheat plants. Meanwhile, infection by *F. graminearum* could preferentially trigger L-TRP’s conversion into serotonin and even phytomelatonin via tryptamine in wheat heads as well. Lower concentrations of them have been shown to stimulate IAA accumulation or mimic IAA to promote plant growth. However, upon that hardly provides sufficient information for regarding alternative methods of controlling scab epidemics. In combination with dissecting IAA biosynthetic pathways using genetic approaches exhibits many difficulties, we thus highlight that ongoing efforts should focus more on identifying the fungal effectors involved in extensive IAA accumulation in cereals in order to understand their potential roles in wheat–*F. graminearum* interactions. Advancements in molecular breeding programs will further accelerate the application of these molecular targets, allowing for the development of more scab-resistant wheat cultivars and resulting in the effective and environmentally friendly suppression of scab epidemics.

## Introduction

Phytohormones are usually defined as organic low-molecular-weight substances in plant tissues that are essential for orchestrating almost every aspect of the developmental process during plant growth, even triggering adaptive responses to diverse abiotic and biotic stresses (Waadt et al. 2022). Among them, auxin is a key phytohormone that promotes cell division, elongation, and differentiation at low concentrations in the main root and shoot as well as in new lateral organs during plant growth (Zhao 2010; Ljung 2013). In addition, accumulating experimental evidence has revealed that diverse microorganisms associated with plants as symbionts or parasites, including bacteria and fungi, can utilize diverse pathways to accumulate the auxin (Tudzynski and Sharon 2002; Tsavkelova et al. 2006; Reineke et al. 2008). Indole-3-acetic acid (IAA) is the major naturally occurring auxin in the interactions between plants and microorganisms (Simon and Petrášek 2011; Enders and Strader 2015; Damodaran and Strader 2019). In particular, infection with *Fusarium graminearum* Schwabe (*Gibberella zeae* (Schwein.) Petch) triggers significant accumulation of IAA in wheat head tissues during the first few days of infection (Qi et al. 2016). The extensive accumulation of IAA in plant tissue might enhance the virulence of the pathogenic fungus *F. graminearum* and subsequently accelerate its infection in the wheat rachis (Cohen et al. 2002). Our latest review carefully summarizes and discusses the possible biological roles of the extensive accumulation of IAA in host tissues, including its biological roles in fine-tuning the defenses of wheat plants and promoting plant growth (Luo et al. 2021). The findings suggest that the repression of auxin accumulation and its mediated signaling cascades during fungal infection in wheat heads is an alternative measure for suppressing scab epidemics in wheat-producing areas and guaranteeing global food security.

Genomic studies have predicted that the genomes of both *F. graminearum* and wheat carry multiple candidate genes that contribute to auxin accumulation in cereals during fungal infection (DesRoches 2012; Su et al. 2021). Meanwhile, additional experimental evidence regarding the prominent causal agents of wheat scab and closely related species reveals that they are capable of IAA biosynthesis upon external feeding with L-tryptophan (L-TRP) and its metabolized intermediates (Tudzynski and Sharon 2002; DesRoches 2012; Tsavkelova et al. 2012; Luo et al. 2016). Recent advances in comparative genomic approaches and reverse genetic approaches have greatly contributed to our understanding of the genetic dissection of auxin biosynthetic pathways in *F. graminearum*. In past decades, extensive studies have typically focused on unraveling more details regarding auxin biosynthesis pathways in *Arabidopsis thaliana* L., which would significantly increase our understanding of the molecular pathways of auxin biosynthesis in wheat plant. In addition, the genome sequences of common wheat were released, providing broad insights into the molecular mechanisms of interactions between *F. graminearum* and wheat mediated by fungus-elicited auxin. Due to various molecular breeding techniques being successfully deployed into wheat cultivar improvement programs (Wang et al. 2020, 2022a; Liu et al. 2021), the substantial advances in the molecular breeding field will enable the application of gene silencing approaches to develop novel cereal cultivars with durable scab resistance through the suppression of IAA accumulation after a fungal invasion. Thus, in this review, we summarize and discuss the currently available knowledge and ongoing research on the putative and preference of auxin accumulation pathways in wheat heads after *F. graminearum* invasion.

## Putative auxin biosynthesis pathways in *F. graminearum*

The experimental evidence in plants demonstrates that the essential amino acid L-tryptophan is the predominant building block for IAA biosynthesis, for which there are both L-TRP-dependent and -independent pathways (Woodward and Bartel 2005; Spaepen et al. 2007). Related studies have greatly contributed to our understanding of auxin synthetic pathways in phytopathogens. They have demonstrated that *F. graminearum* usually adopts L-TRP-dependent pathways during its infection process or in feeding experiments. For instance, after directly feeding *F. graminearum* (virulent strain DAOM 180378, Canadian Fungal Culture Collection, AAFC, Ottawa, ON, Canada) with 2 mM L-TRP, IAA was detected in cultural filtrates within 24 h (hrs) (DesRoches 2012). While our previous feeding experiment revealed that another strain of *F. graminearum* (virulent strain DAOM 233423) was unable to produce detectable IAA during the entire 24 h time-course when 2 mM L-TRP was supplied, indole-3-ethanol (tryptophol) was transiently detected (Luo et al. 2017). Because the cultural filtrates from both strains contained distinct indolic compounds when the cells were supplied with L-TRP individually, in combination with the experimental evidence that IAA was detected when the L-TRP metabolite intermediates were supplied, in this section, we focus more on recent progress regarding the possible IAA biosynthesis pathway in *F. graminearum* with L-TRP metabolite intermediates.

The major well-studied tryptophan-dependent pathways for IAA biosynthesis in microorganisms are named according to their first committed intermediates, including the indole-3-acetamide (IAM), indole-3-pyruvate (IPA), indole-3-acetaldehyde (IAAld), indole-3-acetonitrile (IAN), indole-3-acetaldoxime (IAOx), and tryptamine (TAM) pathways (Patten and Glick 1996; Duca et al. 2014). These committed intermediates can be converted directly to IAA or to appropriate precursors of IAA synthesis in many *Fusarium* species (Tsavkelova et al. 2012; Luo et al. 2016). For instance, based on genomic studies of the fungus, more than 100 candidate genes that are putatively associated with the 14 enzymatic reactions within the proposed pathways for IAA biosynthesis have been predicted in *F. graminearum* (DesRoches 2012). Meanwhile, as described previously, we previously demonstrated that *F. graminearum* can produce IAA through several indolic intermediates, including IPA, TAM, and its derived compound IAN (Luo et al. 2016).

Because the possible intermediates and the genes encoding enzymes associated with the IPA pathway have been found to be present mostly in *Fusarium* species, this pathway is considered the dominant pathway for auxin biosynthesis (Patten and Glick 1996; Duca and Glick 2020). To initiate this pathway, L-TRP is transformed into IPA by aminotransferase enzymes that are putatively encoded by the candidate genes *FGSG_01285* or *FGSG_07188* (DesRoches 2012). After that, IPA can also undergo a decarboxylation process that depends on IPA decarboxylase (encoded by the candidate genes *FGSG_01086*, *FGSG_09834*, *FGSG_10446*, *FGSG_13946*, *FGSG_16351*, etc.) to form IAAld, which is a crucial intermediate for the IPA and TAM pathways (DesRoches 2012; Duca and Glick 2020). This intermediate can then undergo a reaction together with IAAld dehydrogenase (putatively encoded by *FGSG_02273*, *FGSG_05831*, *FGSG_00979*, *FGSG_02392*, *FGSG_02296*, etc.), resulting in the further generation of IAA (DesRoches 2012). Meanwhile, it was demonstrated that IAAld can be transformed into tryptophol by aldehyde reductase (DesRoches 2012), putatively suggesting that directly feeding the *F. graminearum* (virulent strain DAOM 233423) with 2 mM L-TRP would only produce a higher amount of tryptophol (Luo et al. 2017). Moreover, previous studies by ourselves and others also revealed that IPA could be spontaneously transformed into auxin in in vitro experiments (Luo et al. 2016). Therefore, it is hard to determine the importance of IPA pathways in the accumulation of fungus-elicited IAA and the exact ratio of fungus-elicited to spontaneously transformed IAA.

In addition, the TAM pathway is more complex, as it involves more possible intermediates (DesRoches 2012). Initially, L-TRP undergoes a decarboxylation reaction catalyzed by L-TRP decarboxylase (TDC, putatively encoded by the candidate genes *FGSG_03391* or *FGSG_05295*) to form TAM (Patten and Glick 1996; DesRoches 2012). The TAM intermediate then undergoes an oxidative de-amination reaction to form IAAld, usually catalyzed by amine oxidase (putatively encoded by the candidate genes *FGSG_04621*, *FGSG_01758*, or *FGSG_12647*), and then, IAAld is putatively transformed through the IPA pathway to form IAA or tryptophol, as previously described (Patten and Glick 1996; DesRoches 2012). Accordingly, the TAM pathway through IAAld might play a less crucial role in the accumulation of fungus-elicited IAA. In another branch of the TAM pathway, TAM undergoes a hydroxylation reaction catalyzed by flavin monooxygenase (putatively encoded by the candidate genes *FGSG_00712*, *FGSG_03417*, *FGSG_07189*, or *FGSG_11492*) to form N-hydroxyl tryptamine (N–OH-TAM), after which the substance is further oxidized to IAOx (Patten and Glick 1996; DesRoches 2012). Meanwhile, L-TRP can also be directly transformed into IAOx by cytochrome P450 enzymes (putatively encoded by the candidate genes *FGSG_08079*) (Patten and Glick 1996; DesRoches 2012). Subsequently, this intermediate undergoes a deamination reaction to produce IAN. Finally, IAN is catalyzed by nitrilase (NIT, putatively encoded by the candidate genes *FGSG_00051*, *FGSG_00821*, *FGSG_01698*, etc.) to form IAA (Patten and Glick 1996; DesRoches 2012). For instance, the high-performance liquid chromatography (HPLC) chromatograms of the filtrates from *F. graminearum* liquid cultures treated with either 0.2 mM TAM or IAN in a feeding experiment revealed that IAA was detected at 6 h after sampling (Luo et al. 2016). These findings suggest that *F. graminearum* can adopt TAM-dependent pathways to biosynthesize the IAA, either via IAN or IAAld as the intermediates, and there are shared enzymatic reactions with other pathways.

The IAM pathway always involves only two enzymatic reactions, using a monooxygenase and a hydrolase enzyme to form auxin from L-TRP (Kochar et al. 2011). In the mono-oxygenase reaction, L-TRP is catalyzed into IAM through the addition of oxygen from molecular oxygen, and then hydrolase enzymes catalyze the hydrolysis at the carbonyl center of IAM and remove ammonia to form IAA (Patten and Glick 1996; Tsavkelova et al. 2012; Duca et al. 2014). It has been demonstrated that phytopathogenic symptoms during fungal invasions are mostly linked to the IAM pathway (reviewed in Duca et al. 2014), and this pathway has been reported in other *Fusarium* species, while genomic studies of *F. graminearum* have predicted that it is missing the required genes for this pathway (DesRoches 2012; Tsavkelova et al. 2012). Moreover, the HPLC chromatograms of filtrates from cultures treated with 0.2 mM IAM in a feeding experiment revealed that only an IAM peak was present during a 24 h time-course, which suggested that the IAM pathway was not used by *F. graminearum* for the biosynthesis of IAA (DesRoches 2012).

**Fig. 1:**
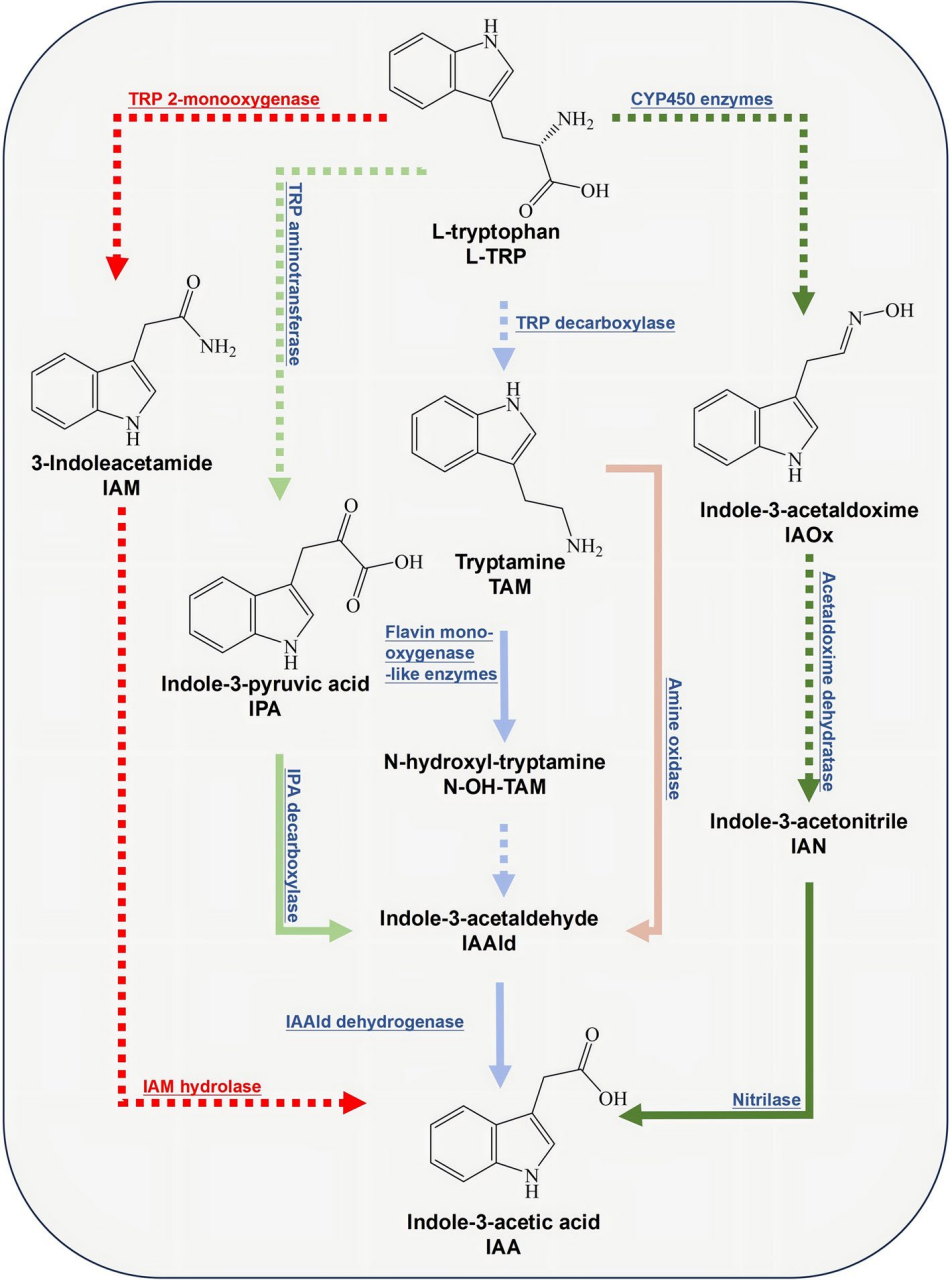
Schematic diagram of the putative L-tryptophan-dependent enzymatic pathways for IAA biosynthesis in phytopathogens. Among them, the tryptamine (TAM) and indole-3-pyruvate (IPA) pathways are shown in *Fusarium graminearum*, while the indole-3-acetamide (IAM) pathway is not present in *F. graminearum* but is present in other related pathogen species. The predicted enzymatic reactions are listed on the arrow of the figure. Solid arrows—conversions observed in or deduced from experimental evidence; dotted arrows—steps predicted from the literature

Altogether, both the experimental evidence from feeding experiments and genome analysis revealed that *F. graminearum* could utilize the intermediates from the IPA and TAM pathways, but not the IAM pathway, to biosynthesize IAA. There are redundant and shared enzymatic reactions among the IAA biosynthetic pathways and the steps needed to synthesize IAA (Fig. 1). However, the potential functions of most candidate genes associated with IAA biosynthesis have not been characterized using reverse genetic approaches, which has significantly hampered the development of effective and environmentally friendly Fusarium head blight (FHB) controls using molecular targets involved in IAA biosynthesis. Therefore, it is necessary to characterize the exact molecular networks of IAA biosynthetic pathways in *Fusarium* species.

## Tryptophan-dependent auxin biosynthetic pathways in plants

Since the chemical identification of the predominant natural auxin, its potential role in plant growth has been studied for nearly 100 years, yet knowledge of endogenous plant biosynthetic pathways remains elusive (Zhao et al. 2002; Finet et al. 2013; Batista-Silva et al. 2024). Although previous studies have proposed multiple pathways for the biosynthesis of IAA, including several tryptophan-dependent pathways and a tryptophan-independent one, the genetic dissection of IAA biosynthesis has proven difficult because few positive IAA-deficient mutants have been identified (Cohen et al. 2003). In comparison, the molecular networks of IAA biosynthesis pathways in pathogenic fungi are well documented, and the candidate genes associated with IAA biosynthesis have been commonly and rapidly determined with reverse genetic approaches (Tsavkelova et al. 2012). There are two possible explanations: (*i*) The genomes of microorganisms are smaller than those of most crops, and it is easy to manipulate deletion or even create double- or triple-gene knockout mutants of candidate genes and complementation transformants. More importantly, these genes usually play crucial roles in pathogenesis. Thus, IAA-deficient strains hardly exhibit inactivation traits, and we could further analyze their biological traits in feeding and inoculation experiments. (*ii*) Advancements in detection and analytical technologies, such as HPLC and gas chromatography/mass spectrometry, have enabled the accurate identification of L-TRP-derived or metabolized compounds in cultural filtrates of microorganisms using external feeding conditions.

Most of our knowledge on the pathways of IAA biosynthesis and degradation in plants comes from scientific studies on Arabidopsis mutants (Zhao 2010). The application of technologies such as isotope labeling, gene silencing, and genome sequencing and editing has been attributed to the advancement of studies on the genetic dissection of small molecule or secondary compound biosynthesis in diverse plants. Similarly, direct feeding plants with labeled L-TRP yields labeled IAA, and diverse L-TRP metabolite intermediates had been detected in plant tissues at a given development stage, indicating that plants could adopt tryptophan-dependent pathways to biosynthesize IAA (Gao and Zhao 2014). However, few IAA biosynthesis genes have been identified and characterized in plants. This is probably because dissecting IAA biosynthesis using genetic approaches exhibits many difficulties (Zhao et al. 2002). For instance, studying auxin biosynthesis is made difficult by the fact that loss-of-function mutants are either sterile or have intrinsic heterogeneity in their phenotypes, which limits their utility for examining auxin biosynthetic pathways. Meanwhile, these mutants have to rescue this situation by activating alternative auxin biosynthetic pathways (Zhao et al. 2002). Thus, the overexpression or knockdown of the only enzyme conclusively shown to be involved in one of the branches of auxin biosynthesis could be the dominant method for the genetic analysis of IAA biosynthesis networks.

The *YUCCA* genes were the first IAA biosynthesis gene family to be identified and characterized, probably because they encode the flavin monooxygenase (FMO)-like enzyme or indole‐3‐pyruvate monooxygenase (Zhao et al. 2001), one of the important nodes of the IPA and TAM pathways associated with IAA biosynthesis in plants. The IPA pathway in Arabidopsis similarly stands as a major and well-characterized pathway (Patten and Glick 1996). To initiate this pathway in plants, L-TRP is similarly converted into IPA by the enzyme tryptophan transferase/SAV3 (TAA1, Tryptophan Aminotransferase of Arabidopsis 1/Weak Ethylene Insensitive 8/Shade Avoidance 3/Cytokinin Induced Root Curling 1), and the YUCCA enzyme has been proposed to subsequently catalyze the conversion of IPA into IAA (Stepanova et al. 2011; Batista-Silva et al. 2024). This is supported by the fact that the phenotypes of *YUCCA1* overexpression in *taa1/sav3* background deletion exhibited weakened auxin production, while the wild type (WT) background leads to dramatic auxin overproduction phenotypes (Won et al. 2011). Thus, *YUCCA* genes putatively encode indole‐3‐pyruvate monooxygenase for IAA biosynthesis through the IPA pathway. Studies using integrated metabolo-transcriptomics conducted in wheat inoculated with *F. graminearum* have supported this idea. For instance, the candidate gene *TraesCS1D01G238100* has been putatively predicted to be *TaTAA1*, which was found to be significantly upregulated in wheat tissues, trigger the accumulation of IPA from L-TRP, and subsequently be transformed into the intermediate IAAld by indole‐3‐pyruvate monooxygenase (YUCCA, putatively encoded by wheat candidate genes *TraesCS3D01G030500*). The RNA-seq data in that study demonstrated that IAAld was subsequently converted into IAA by indole‐3‐acetaldehyde oxidase (AAO, putatively encoded by wheat candidate genes *TraesCS2D01G248700* and *TraesCS2A01G246300*), and aldehyde dehydrogenase (ALDH, putatively encoded by wheat candidate genes *TraesCS4A01G219900*, *TraesCS7A01G210100*, *TraesCS6D01G222000*, *TraesCS6D01G272400*, *TraesCS2A01G388900*, *TraesCS5A01G213600*, *TraesCS6B01G284600*, etc.), because a multitude of candidate genes involved in this pathway were upregulated (Su et al. 2021). Therefore, the upregulation of the transcription of the *TaTAA1* gene suggests that the extensive accumulation of IAA in wheat head tissues after *F. graminearum* inoculation is predominantly triggered by the IPA pathway.

Early biochemical analysis indicates that the YUCCA enzyme (putatively the FMO-like enzyme in the TAM route) catalyzes the conversion of TAM to form N-TAM, and then N-TAM may be converted to IAOx, a proposed precursor for IAA biosynthesis (Zhao et al. 2001). However, the accumulating experimental evidence from high-resolution analytical techniques in combination with reverse genetic approaches and in vitro assays has revealed that *YUCCA* genes do not play a major role in IAOx production (Sugawara et al. 2009). For instance, genetic experimental evidence revealed that IAOx levels were not significantly altered in a *yuc1 yuc2 yuc4 yuc6* quadruple mutant, while *cyp79b2 cyp79b3* double mutants did not exhibit any detectable IAOx upon the in vivo feeding of labeled indoles to Arabidopsis. This suggests that the plant is not dependent on the TAM but L-TRP as a substrate for the synthesis of IAOx (Sugawara et al. 2009). Meanwhile, no orthologs of *CYP79B* genes have been reported in the genomes of many Poaceae crops, such as rice and maize (Sugawara et al. 2009). Accordingly, wheat inoculated with *F. graminearum* could not trigger the extensive accumulation of IAOx. However, inoculation with *F. graminearum*, pest attacks, or direct phytohormone treatment significantly upregulated the transcript of the *TraesCS2A01G516500* gene (putatively predicted to be *TaTDC*) and induced the accumulation of TAM in plant tissue (Brauer et al. 2019; Su et al. 2021; Yan et al. 2023). TAM could be the precursor of IAAld or IAN in diverse plants, but few studies have focused on elucidating this. Moreover, as described previously, the FPKM expression values of candidate genes predicted to encode the AAO and ALDH enzymes were found to be upregulated in wheat tissues after inoculation with *F. graminearum* (Su et al. 2021). Additionally, the wheat genome harbors the candidate genes corresponding to NIT. Thus, we cannot exclude the possibility that the TAM pathway plays a crucial role in IAA accumulation in wheat tissues. To better understand the TAM metabolites’ role in mediating fungus–plant or pest–plant interactions, we focus more on discussing the TAM-dependent IAA accumulation pathways in the following section.

Although the IAM pathway is the best-characterized pathway of IAA production and was initially considered a microorganism-specific pathway, accumulating experimental evidence has revealed that this route is also found in diverse plants (Lehmann et al. 2010). For instance, in silico analyses revealed that the orthologs of candidate Arabidopsis IAM-hydrolase (*AtAMI*) have wider distribution in Poaceae crops (Sánchez-Parra et al. 2014; Batista-Silva et al. 2024). Further functional analyses of *AMI* in five selected plant species demonstrated that recombinant amidases could convert IAM into IAA, while its preferred substrate is phenyl-2-acetamide, as it showed the highest conversion rate when compared with several related substrates (Sánchez-Parra et al. 2014). Meanwhile, several plant species, e.g., rice and maize, have been identified to contain IAM as a natural constituent in their tissue (Sánchez-Parra et al. 2014). Although it was reported that IAM could be transformed from IAOx in Arabidopsis (Sugawara et al. 2009), it remains unclear how exactly IAM is produced in crops. A possible explanation for how IAM is synthesized from L-TRP is that tryptophan-2-monooxygenase (*iaaM*) in plant pathogenic bacteria or fungi enables the accumulation of IAM in plant tissue during the infection process (Pollmann et al. 2009). This is probably because genomic studies found non-homologs of microorganism tryptophan-2-monooxygenase in the higher plant kingdom, and it is the required gene for this pathway.

**Fig. 2:**
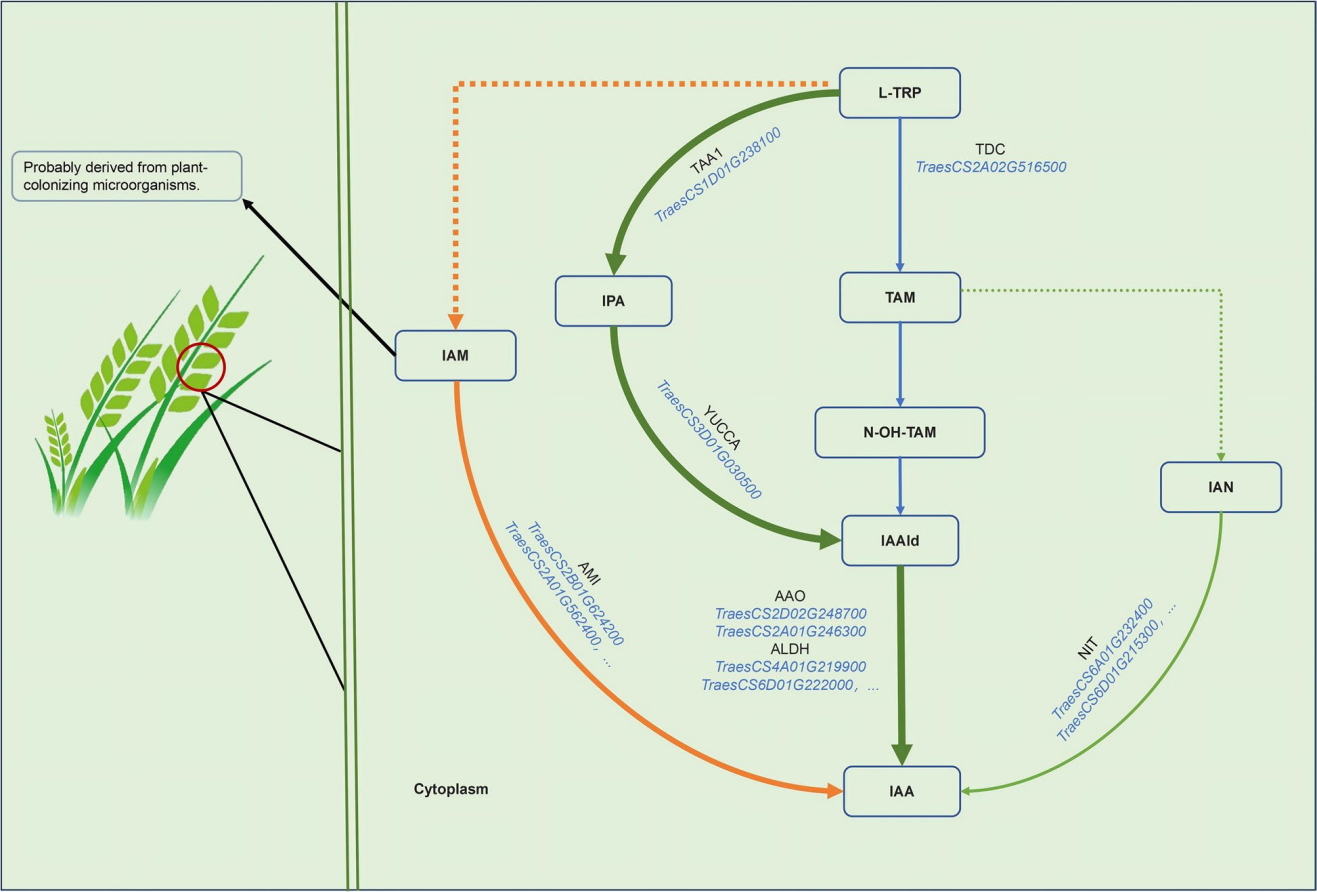
Proposed pathways of L-tryptophan (L-TRP)-dependent IAA biogenesis in wheat plants. There are three pathways, including the indole-3-pyruvate (IPA), tryptamine (TAM), and indole-3-acetamide (IAM) pathways. The IPA pathway could be the dominant IAA biosynthetic pathway, and the candidate genes predicted to encode the corresponding enzymes involved in this pathway were identified. The TAM pathway can proceed through either indole-3-acetonitrile (IAN) or indole-3-acetaldehyde (IAAld) to form IAA. The accumulation of IAM in wheat tissue is seemingly triggered by infections of microorganisms, probably because wheat’s genome does not harbor the L-TRP monooxygenase, while the fungus has it. Dashed lines indicate the assumed reaction steps for which the corresponding enzymes have yet to be identified. N–OH-TAM, N-hydroxyl tryptamine; TDC, L-TRP decarboxylase; TAA1, Tryptophan Aminotransferase of Arabidopsis 1; YUCCA, indole‐3‐pyruvate monooxygenase; AAO, Indole‐3‐acetaldehyde oxidase; ALDH, Aldehyde dehydrogenase; AMI, amidases 1; NIT, nitrilase

Altogether, the above studies suggest that the inherent IAA biosynthetic pathways derived from IPA, TAM, and IAM enable wheat plants to elevate the level of IAA in their tissues (Fig. 2). Furthermore, the inoculation of fungal attackers usually triggers extra accumulation of IAA in the host tissue via fungus-elicited IAA and/or defense hormone signals. Thus, the following section will focus on unraveling these issues.

## Fungal invasion preferentially alters the pathways of L-TRP metabolism and auxin accumulation in wheat heads

Our previous studies demonstrated that directly supplying the phytopathogen *F. graminearum* with the substrates of IAA biosynthesis in a feeding experiment led to the detection of a similar level of auxin as its substrates, while fungal infection led to the accumulation of approximately 400 times as much IAA in Roblin cultivar wheat head tissues (Luo et al. 2016; Qi et al. 2016). As discussed in the last two sections, the level of IAA accumulation in wheat heads during the fungal infection process could be influenced by pathways present in both fungi and plants. Owing to L-TRP and its derived compounds play a dominant role in auxin biosynthesis in plants and fungi, thus their levels could be elevated after fungal invasion (Paranidharan et al. 2008; Kumaraswamy et al. 2011; Pasquet et al. 2014; Brauer et al. 2019). For instance, previous studies have demonstrated that infection with *F. graminearum* leads to the increased biosynthesis of L-TRP and derived compounds in wheat and *Brachypodium distachyon* (Pasquet et al. 2014). In addition, the expression profiles 4 days post fungal inoculation (dpi) obtained from microarray data revealed that *F. graminearum* infection in either scab-susceptible or -resistant wheat cultivars significantly upregulated the genes associated with indole-3-glycerol phosphate synthase in wheat plants (Brauer et al. 2019). This enzyme plays a dominant role in the conversion of 1-(2-carboxyphenylamino)-l-deoxyribulose-5-phosphate to form tryptophan in combination with tryptophan synthase alpha and beta subunit (Richter et al. 2021). However, our previous in vitro feeding experiment revealed that, when *F. graminearum* cultures were supplied with L-TRP, indole tryptophol was immediately detected, and no auxin was detected during the entire feeding experiment (Luo et al. 2017). Meanwhile, during the early fungal infection stage, *F. graminearum* slightly upregulated the relative expression level of the *FGSG_09834* gene, which encodes fungus IPA decarboxylase, and the transcripts of *FgTDC* candidate gene *FGSG_05295* at all sampling timepoints in the spikelet tissues of the susceptible Roblin wheat cultivar (Brauer et al. 2019). However, neither of them was expressed under experimental feeding conditions, according to the expression profiles of *F. graminearum* when 2 mM L-TRP was directly supplied in culture (DesRoches 2012). These results suggest that, during the early stage of infection, *F. graminearum* can utilize L-TRP and its derived compounds in wheat heads through the pathways described in the previous section to biosynthesize IAA. Furthermore, environmental alterations in plant tissues, especially those induced by plant immune systems after biotic attacks, provide an important clue for characterizing the exact pathways of auxin accumulation in wheat plants triggered by *F. graminearum* infection.

Following an attack, plants have to activate their intricate, dynamic, and effective immune defenses to combat or alleviate further damage, a strategy that coevolved with long-term adaptations to resist biotic stresses (Thomma et al. 1998; War et al. 2012). One of the best-understood defense responses in plant immune systems is the accumulation of defense phytohormones, including jasmonic acid (JA) and salicylic acid (SA) (Shigenaga and Argueso 2016). Therefore, the physiological alterations in plant tissues triggered by the accumulation of defense phytohormones might synergistically facilitate the extensive accumulation of IAA in host plant tissues after fungal invasion. Our latest studies demonstrated that either directly spraying MeJA onto foliage or the indirect accumulation of jasmonates (JAs) after aphid preinfestation in plant tissue significantly upregulated the transcript of the *TaTDC* gene in wheat cultivar XN979, and its expression products significantly transform L-TRP to TAM (Yan et al. 2023). Meanwhile, the early stages of *F. graminearum* infection significantly induced wheat plants to accumulate a high level of JA and significantly upregulated the expression of the *TaTDC* gene two and four days post inoculation (over 60-fold) relative to one day after infection in the wheat cultivar Roblin (Brauer et al. 2019). Moreover, some fungal candidate genes of *TDC* and IPA decarboxylase exhibited different expression profiles between the feeding experiment and real inoculation (DesRoches 2012; Brauer et al. 2019). Therefore, the environmental changes triggered by the extensive accumulation of defense phytohormones in plant tissues, especially JA and its derived compounds, could be the crucial inducer for the significant upregulation of the transcription of the *TaTDC* gene and the production of TAM compounds.

Although the latest studies have demonstrated that Arabidopsis plants do not metabolize TAM as the dominant substrate with the aid of YUCCA enzymes for IAA accumulation (Sugawara et al. 2009), TAM and its derived compounds can be transformed into a similar amount of IAA via different biosynthesis pathways in *F. graminearum*, and the candidate genes that are predicted to participate in IAA biosynthesis have been identified in the genome of this fungus (DesRoches 2012; Luo et al. 2016). Subsequently, fungus-elicited IAA through TAM pathways could stimulate the extensive accumulation of a huge amount of auxin in wheat tissues via non-TAM pathways. The experimental evidence obtained from the canola *Brassica napus* and wheat supports this conclusion. For instance, canola seedlings that were treated with IAA significantly upregulated genes involved in the IPA and IAOx pathways of auxin biosynthesis, including the *TAA1, TAR2, TAR3, TAR4, YUCCA,* and *CYP79B2/3* gene families (Liu et al. 2023)*.* Meanwhile, the candidate gene *TaTAA1* was significantly upregulated in the leaf tissue of a Sumai 3 wheat cultivar that was inoculated with different races of *F. graminearum*, including R40, R64, S52, and S66 (Su et al. 2021). Because the pathways of auxin biosynthesis in wheat plants have not yet been completely illustrated, it is hard to describe the exact pathways of auxin accumulation triggered by fungus-elicited IAA and/or the direct conversion of TAM to form IAA in plants.

In addition, our latest results revealed that there is probably a positive relationship between JA treatment and IAA accumulation. For instance, the relative expression of *TaCAD* was found to be significantly suppressed after MeJA application on wheat leaves, and it exhibited the lowest value 6 h post spraying, while aphid infestation significantly increased its transcript at all sampling timepoints (Yan et al. 2023). This gene is always associated with lignin biosynthesis and plays an important role in reinforcing the cell wall, because the accumulation of IAA in plant tissues could loosen the cell wall to stimulate plant growth, and its transcripts are fundamentally suppressed by the elevation of IAA in plant tissues (Bi et al. 2011). Thus, the accumulation of IAA paralleled JA accumulation in plant tissues and its mediated signaling pathways, further altering the plant’s physiological properties to directly benefit from fungal invasions or pest infestations (Downes et al. 2001; Lasík 2003; Ding et al. 2008; Fu and Wang 2011; Zhang et al. 2012). Meanwhile, the direct application of auxins on plant leaves significantly upregulated the relative expression of genes involved in JA biosynthesis, including the *AOS* and *LOX2* genes (Iskender and Staswick 2002). Moreover, our latest study revealed that the direct foliage application of MeJA immediately elevated the above-mentioned JA biosynthesis genes, and both of them reached their highest values 6 h post spraying (Yan et al. 2023). This may suggest that JA or its induced IAA accumulation in wheat tissues could upregulate the relative expression of genes involved in JA biosynthesis. Based on these results, there would be a self-amplifying feedback loop between auxin and MeJA accumulation in different plants under biotic stress, and it is difficult to exactly determine the reasons why the transcript of the JA biosynthesis genes is upregulated.

Apart from that, the rapid and extensive accumulation of hydrogen peroxide (H_2_O_2_) is one of the most important consequences of defense phytohormone accumulation in the tissues of host plants (Chen et al. 1993b, a; Penninckx et al. 1998; Song et al. 2021). As an important indicator and secondary messenger of defense phytohormone-mediated signaling cascades, H_2_O_2_ accumulation would be advantageous for enabling host plants to exhibit system-acquired resistance to withstand the fungal invasion (Johnson et al. 2003; Mou et al. 2003; Makandar et al. 2006; Wu et al. 2012; Kumar 2014). On the contrary, plants trade off the plant immune response triggered by extra H_2_O_2_ accumulation into growth, increasing the activity of the enzymes involved in regulating cellular H_2_O_2_ homeostasis, such as catalase (CAT). For instance, during the seedling post-germinative growth stage, Arabidopsis seedlings elevate the activity of CAT2 to fine‐tune cellular H_2_O_2_ homeostasis and then significantly increase the activity of ACX2/3, which catalyzes the first step of the β‐oxidation pathway in JA biosynthesis and enables plants to accumulate JA in tissues (Liu et al. 2017; Yuan et al. 2017). Accordingly, there would be a self-amplifying feedback loop between JA and H_2_O_2_ accumulation in plants under diverse biotic and abiotic stresses (Gao et al. 2014; Liu et al. 2017; Yuan et al. 2017).

**Fig. 3:**
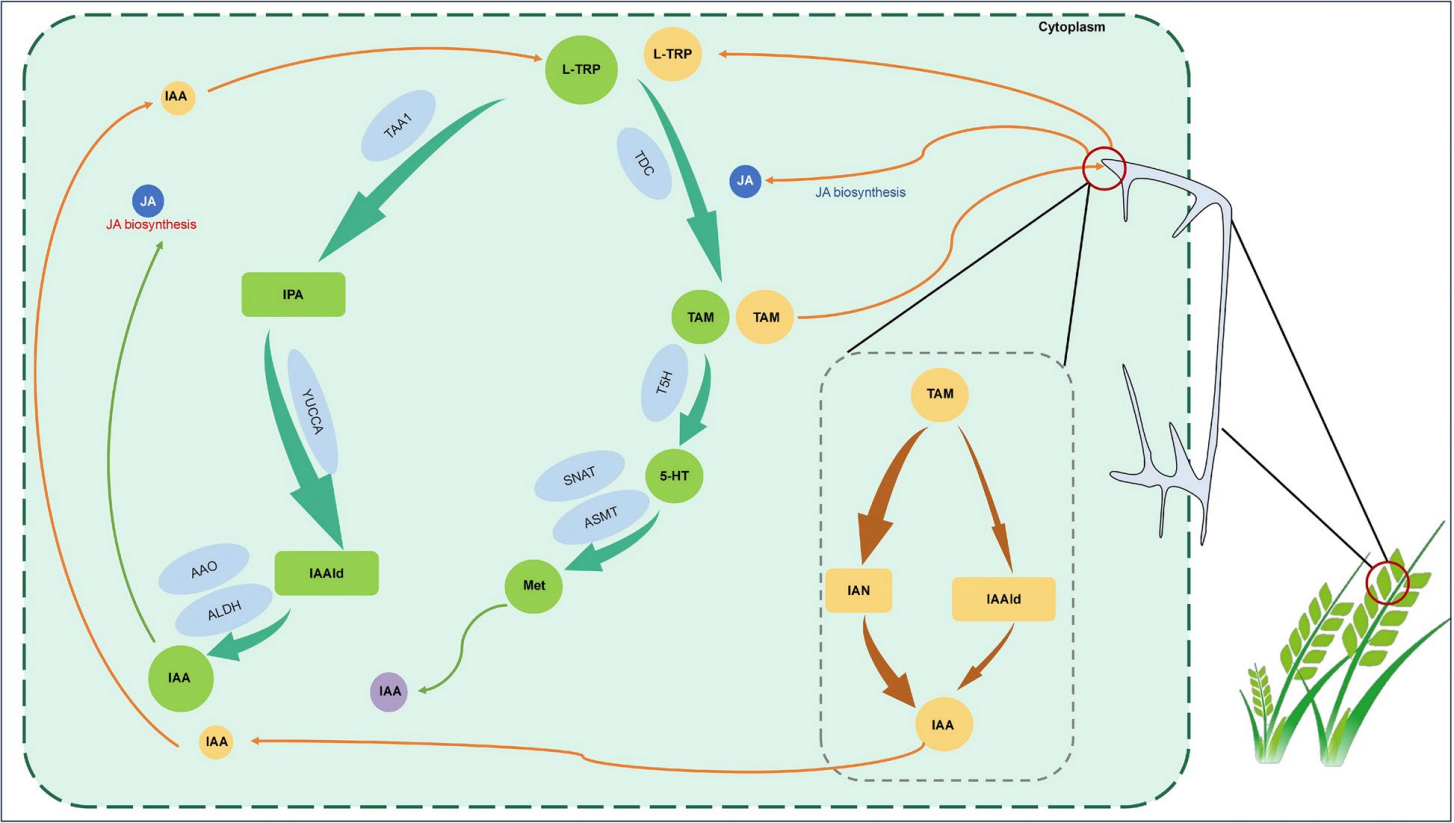
The potential pathways of extensive IAA accumulation during early-stage infection of *Fusarium graminearum*. *F. graminearum* colonization induces L-tryptophan (L-TRP) and jasmonic acid (JA) accumulation, which may result in higher *TaTDC* expression and the accumulation of tryptamine (TAM) in wheat seedlings. The fungus can utilize TAM to biosynthesize fungus-elicited IAA through either indole-3-acetonitrile (IAN) or indole-3-acetaldehyde (IAAld). This elicited IAA can not only induce the wheat plant to upregulate the transcripts of marker genes involved in JA biosynthesis but also preferentially activate the dominant IAA biosynthetic pathway—the indole-3-pyruvate (IPA) pathway—leading to the extensive accumulation of IAA. Meanwhile, TAM can be transformed into serotonin (5-HT) and phytomelatonin (Met), and a lower concentration of Met can stimulate IAA accumulation. The butter color arrows depict the potential roles of the fungus infection in the IAA accumulation in the host tissue, while the green color arrows indicate the IAA accumulation pathways in wheat heads. TDC, L-TRP decarboxylase; T5H, Tryptophan hydroxylase; TAA1, Tryptophan Aminotransferase of Arabidopsis 1; YUCCA, indole‐3‐pyruvate monooxygenase; AAO, Indole‐3‐acetaldehyde oxidase; ALDH, Aldehyde dehydrogenase; SNAT, Serotonin N-acetyltransferase; ASMT, N‐acetylserotonin methyltransferase

Altogether, the exact pathways of extensive auxin accumulation during *F. graminearum* inoculation are probably derived from the IPA pathway of host plants triggered by fungus-elicited IAA via the TAM pathway, and the physiological alterations triggered by JA deposition in hosts induced the transformation of L-TRP into the substrates of fungus-elicited IAA (Fig. 3). However, these cannot fully explain the exact pathways of extensive IAA accumulation in wheat plants damaged by biotic attackers, so more work is required to unravel the IAA biosynthetic molecular network in wheat during the early stage of *F. graminearum* invasion.

## Potential functions of tryptamine and its metabolites associated with auxin accumulation in plants after fungal invasion

As described previously, either MeJA treatment or colonizer attacks could directly upregulate the relative expression of the *TaTDC* gene and then result in the extensive accumulation of TAM in plant tissues. A recent study that adopted the metabolome genome-wide association approach confirmed that TAM is a possible substrate for forming serotonin (5-hydroxy tryptamine, C_10_H_12_N_2_O) and even phytomelatonin (*N*-acetyl-5-methoxytryptamine, C_13_H_16_N_2_O_2_) in wheat plants (Chen et al. 2023). In addition, integrated metabolo-transcriptomics studies have predicted that wheat tissues would accumulate serotonin and even phytomelatonin when inoculated with *F. graminearum* (Su et al. 2021). Despite the lack of direct experimental evidence that serotonin can promote auxin accumulation after fungus infection, the direct application of this compound at lower concentrations on Arabidopsis plants has been shown to stimulate primary root growth and root hair formation in a similar manner to IAA, probably because they share structural similarities and auxin receptors can facilitate serotonin’s transport into plants (Pelagio-Flores et al. 2011; reviewed in Mukherjee 2018; Sun et al. 2025). While higher-dose treatment restricted the plant’s root growth and development, further analysis revealed that serotonin exhibited anti-auxin activity by strongly suppressing the expression of auxin-responsive genes (Pelagio-Flores et al. 2011). Meanwhile, abiotic stress inhibits the seedling growth of sunflower (*Helianthus annuus* L.), partially by impairing auxin signaling, and this coincides with the extensive deposition of serotonin (Mukherjee et al. 2014). This suggests that either biotic or abiotic stresses that induce an upsurge in serotonin can impair auxin biosynthesis, transport, and responses, probably because both serotonin and auxin are derived from the same precursor, L-TRP. In addition, serotonin functions as a reactive oxygen species (ROS) scavenger that confers resistance to further fungal infections (Azizi et al. 2019). For instance, the dramatic increase in serotonin induced after *Magnaporthe oryzae* penetration in rice significantly attenuated or rescued leaf tissue from lesion browning caused by a hypersensitive response (HR). Subsequently, the deposition of an oxidized form of serotonin has been detected at the HR lesion sites (Hayashi et al. 2016). Moreover, exogenous treatment with serotonin can preserve the cellular integrity of plant tissues surrounding the fungus inoculation sites and suppress the further growth of fungal hyphae in rice leaf tissues (Ishihara et al. 2008). Thereafter, an HR often induces broad-spectrum systemic enhanced resistance–systemic acquired resistance (SAR) to pathogenic infection, which is always dependent on the deposition of SA in plant tissues. Because either an HR or SAR inevitably confers a yield penalty, phytopathogens, especially in their biotrophic stage, adopt the extensive deposition of serotonin to trade off the plant’s immune responses to growth. Furthermore, serotonin and SA are derived from the same precursor, chorismite; thus, the extensive deposition of serotonin would suppress the accumulation of SA and its mediated responses. Similarly, insect attackers preferentially alter the TAM conversion pathways to terminate at the stage of significant serotonin accumulation, which would directly stimulate the feeding behavior of insects and antagonize SA-mediated responses in plants (Lu et al. 2018). Altogether, the deposition of serotonin during fungus invasion can exhibit both positive and negative regulatory effects on physiological alterations mediated by auxin signaling, depending on its concentration.

Compared to serotonin, a low concentration of phytomelatonin can promote auxin biosynthesis and transport, while high concentrations can inhibit auxin accumulation (reviewed in Mukherjee 2018; Sun et al. 2025). However, few studies have focused on characterizing the underlying molecular mechanisms. This is probably because phytomelatonin has similar functions to IAA in terms of promoting plant growth at lower concentrations. Thus, it is hard to determine whether phytomelatonin-elicited IAA is produced in wheat plants under a low concentration of phytomelatonin during the early stage of *F. graminearum* infection by *F. graminearum*. Additionally, the extensive accumulation of phytomelatonin could trigger plant defenses against diverse phytopathogens. For instance, transcriptomic and metabolomic analyses revealed that phytomelatonin accumulation mediated by the phytomelatonin biosynthesis gene *TaASMT3* made wheat resistant to stripe rust (Jiang et al. 2025). Meanwhile, Arabidopsis plants with mutated serotonin N-acetyltransferase (SNAT) exhibited decreased phytomelatonin and SA levels, resulting in susceptibility to avirulent pathogens (Lee et al. 2015). This suggested that the phytomelatonin-mediated plant defenses may depend on SA signaling pathways to protect against the fungal colonizers. This conclusion had been supported by the experimental evidence that the exogenous application of phytomelatonin could coordinate with defense phytohormone-dependent pathways, and subsequently trigger the upregulation of marker genes involved in these defense responses to combat phytopathogens (Lee et al. 2015; Shi et al. 2015a, b; Lee and Back 2017; Chen et al. 2020). Moreover, it has been confirmed that phytomelatonin alters the activity of antioxidant enzymes to enable plants to withstand damage caused by abiotic stresses, including heavy metals, salination, coldness, and UV radiation (Shi and Chan 2014; Reiter et al. 2015; Shi et al. 2015a, b; Liang et al. 2017; Zhang et al. 2017; Li et al. 2021). Because IAA in wheat heads is highest after *F. graminearum* inoculation (Fig. 3), combined with the fact that low concentrations of TAM metabolites can promote auxin biosynthesis, phytomelatonin-elicited IAA probably accounts for a small proportion of total IAA.

Therefore, comprehensively interpreting the relationship between physiological alternations, phytohormone crosstalk pathways, and IAA accumulation in plant tissue is necessary to decipher the pathogenicity of *F. graminearum* and to develop scab-resistant cultivars.

## Silencing the genes leading to IAA accumulation probably strengthens crop scab resistance

According to suggestions from the Chinese National Agro-Tech Extension and Service Center, chemical control is still the most important measure for suppressing the epidemic of FHB disease in agricultural production, as it can effectively repress the extended growth of the fungus and mycotoxin accumulation in wheat heads in a short time. However, it is apparent that chemical spraying gives rise to a multitude of problems, including environmental pollution, public health issues, and the continuously increasing pathogenic varieties of fungus, subsequently leading to the resurgence of pests. In addition, during the wheat heading and flowering stage, the key period of wheat scab prevention and control, cloudy and rainy days are common, which makes it impossible to quickly and effectively prevent and control the disease with chemicals, further aggravating the scab epidemic. Compared with chemical control measures, the wide-ranging cultivation of scab-resistant wheat cultivars would be a more cost‐effective and environmentally friendly measure for suppressing the epidemic of FHB disease. During the past few decades, considerable progress has been made in genetically engineered wheat improvements to strengthen their biotic resistance. However, the breeding and development of scab-resistant wheat cultivars still face a diverse range of challenges, probably because most of the scab resistance traits in wheat germplasms are controlled by quantitative trait locus (QTL) alleles (Miedaner et al. 2006; Xue et al. 2010). It has been demonstrated that the performance of most QTLs is easily affected by environmental factors, resulting in an inability to stably exhibit traits associated with scab resistance (Bernardo 2008). Therefore, stacking more different scab resistance QTLs will be necessary to strengthen the available wheat varieties with resistance to FHB (Luo et al. 2023a). However, the experimental evidence has revealed that less‐than‐additive effects are exhibited when compared with progeny carrying the individual beneficial QTL alleles (Eshed and Zamir 1996; Marcel et al. 2007; Bernardo 2008). Accordingly, the introgression of scab resistance genes or QTLs into elite common wheat varieties to strengthen their resistance to FHB still requires substantial efforts, and only a small portion of resistance alleles have been transferred into the common wheat background, which cannot fulfill the demand for scab-resistant wheat cultivars in agricultural production.

Apart from directly strengthening the scab-resistant of wheat cultivars, knocking down the genes associated with fungus pathogenicity or scab susceptibility in elite wheat cultivars could be an alternative way to introduce scab-resistant traits. Recently, RNA interference (RNAi)-mediated gene silencing approaches, including directly spraying dsRNA-induced gene silencing (SIGS), virus‐induced gene silencing (VIGS), and host‐induced gene silencing (HIGS) agents, have been widely adopted and have successfully suppressed scab epidemics (Koch et al. 2013; Cheng et al. 2015; Qi et al. 2019; Wang et al. 2020; Liu et al. 2021). Among them, direct spraying and virus-induced methods are considered transient silencing approaches, while host-induced methods could enable wheat breeders to develop cultivars with durable scab resistance. Our previous studies comprehensively reviewed the application of these RNAi-mediated gene silencing approaches when determining the potential biological roles of the wheat genes involved in either scab susceptibility or resistance (Luo et al. 2023a). Based on these available data, we suggest that transient silencing approaches could be a suitable strategy for silencing fungus proteins associated with extensive IAA accumulation post fungus inoculation, such as those encoded by the wheat endogenous genes *TaTAA1*, *TAR2, TAR3, TAR4, YUCCA*, etc. Meanwhile, silencing them could strengthen the scab resistance of elite wheat cultivars to withstand even damage caused by herbivorous insects. Because IAA probably plays a dominant role in facilitating normal plant growth, these genes cannot be mutated through genome editing and knockout. Compared with VIGS, SIGS could more suitable for suppressing plant endogenous genes associated with IAA accumulation after fungal invasion in agricultural production. In addition, the SIGS strategy has been proven to effectively suppress the transcription of corresponding phytopathogenic genes (Koch et al. 2013). Moreover, an increasing number of firms can provide dsRNA synthesis services, which would enable cereal producers to easily control damage caused by different biotic stresses. These findings suggest that SIGS could provide an alternative measure for precisely controlling the FHB epidemic; however, numerous factors, including but not limited to target gene selection, dsRNA uptake mechanisms, barriers to dsRNA uptake, dsRNA/siRNA design parameters, dsRNA dose and size, application method, delivery strategy, and environmental stability, may affect the efficacy of exogenously applied RNA molecules in triggering successful RNAi in plant–fungus interactions. Therefore, the application of transient gene silencing approaches is not sufficient to suppress epidemics of scab in cereal crop production.

Although the HIGS approach has not been used to knock down the wheat endogenous genes that contribute to IAA accumulation, this approach could precisely suppress the fungal genes encoding the effectors that are secreted during the early stage of infection, even those related to extensive IAA or JA accumulation in host plants, enabling the development of wheat cultivars with durable scab resistance. The accumulating experimental evidence has revealed that *F. graminearum* infection could simultaneously activate the SA- and JA-mediated defense responses, playing crucial roles in combatting further damage, and JA usually suppresses the defenses mediated by SA through IAA accumulation or directly suppresses the genes associated with SA accumulation and its mediated defense responses. Thus, the application of the HIGS approach to silence the genes targeting the fungal avirulence or effector proteins associated with extensive IAA or JA accumulation will therefore suppress JA and IAA accumulation and their mediated responses. This would subsequently strengthen SA-mediated defense responses to withstand biotic stresses. To successfully establish a parasite relationship and suppress host defense responses, pathogen-secreted effectors usually play critical roles during fungus and host interactions. For instance, *F. graminearum* secretes more than 350 effectors into wheat apoplast or plant cells to manipulate plant physiology and the immune system (Rocher et al. 2022). In recent decades, plenty of effector-coding genes and their molecular targets have been identified and characterized. Among them, effectors encoding genes including *FGSG_01831* and *FGSG_03599* could suppress chitin-triggered ROS generation and BAX-induced cell death (Hao et al. 2019). However, this study did not determine the relationship between JA or IAA accumulation and the symptom of suppressing ROS generation and cell death. More substantial work is required to comprehensively identify the relationship between the pathogenicity of pathogen effectors and JA or IAA accumulation. Overall, the large-scale cultivation of scab-resistant wheat cultivars developed with HIGS breeding approaches and the timely spraying of dsRNA that targets the fungal avirulence or effector proteins associated with extensive IAA or JA accumulation during fungal outbreaks could provide an alternative way to effectively suppress the scab epidemic.

## Concluding remarks

The present review illustrates and summarizes most of the currently available literature on the putative L-TRP-dependent pathways for wheat heads’ IAA accumulation after *F. graminearum* invasion, based on the available knowledge of the endogenous IAA biosynthetic pathways in *F. graminearum* and wheat plants. Meanwhile, the plant’s physiological alterations could trigger a preference for L-TRP conversion and auxin accumulation in wheat heads, L-TRP serotonin and even phytomelatonin via TAM, and lower concentrations of TAM derived compounds, including serotonin and phytomelatonin have been shown to stimulate IAA accumulation or simulate IAA to promote plant growth. However, these pieces of experimental evidence only approximately describe the molecular networks of IAA accumulation during the first few days after inoculation, hardly providing adequate fundamental information regarding alternative methods of controlling scab epidemics. Therefore, future work is necessary to exactly describe the molecular pathways and unravel the significance of extensive IAA accumulation in enhancing the pathogenicity of *F. graminearum.* Furthermore, the fungal genes associated with pathogenicity, mycotoxin production, vegetative growth, sporulation, reproduction divergence, stress tolerance, etc., have been identified and characterized with reverse genetic approaches, including knocking out the gene of interest or even by performing double- or triple-gene knockouts (reviewed in Xu et al. 2022; Luo et al. 2023b). These genes, which are especially effective at the early infection stage, have provided sufficient molecular targets for breeding novel wheat cultivars with durable scab resistance through the HIGS approach.

In addition, identifying more fungal effectors involved in extensive IAA accumulation in cereals is urgent; understanding their potential roles in wheat–*F. graminearum* interactions will enable us to select molecular targets for the suppression of scab epidemics in an effective and environmentally friendly manner. In recent decades, genome editing has emerged as a most promising method in crop cultivar improvement programs to strengthen stress resistance, probably because, in most cases, genome editing does not introduce any yield penalty. Especially, a stepwise protocol for the clustered regularly interspaced short palindromic repeats (CRISPR)/CRISPR-associated endonuclease 9 (Cas9) system-based editing of cereal genomes has been described, which significantly facilitates more accurate targeting in mutagenesis in wheat breeding programs (Shan et al. 2014). For instance, three homoalleles of the wheat transcription factor gene *TaWRKY19* were mutated with the CRISPR/Cas9 system, resulting in enhanced *TaNOX10* gene transcription and promoting its mediation of host plant ROS accumulation, and triple *tawrky19-ko* mutant wheat seedlings exhibited strong resistance to the biotrophic pathogen *Puccinia striiformis f. sp. tritici* (Wang et al. 2022b). These advances provide the potential to effectively mutate the wheat transcription factors targeted by fungal effectors associated with extensive IAA accumulation through CRISPR/Cas9 system-mediated genome editing strategies.

In conclusion, the ongoing advances and efforts regarding molecular breeding approaches will significantly accelerate the application of these potential genetic engineering targets to develop more scab-resistant wheat cultivars, whose large-scale cultivation will further promote the sustainable production of grains and ensure food security worldwide.

## Data Availability

Data sharing does not apply to this article as no datasets were generated or analyzed during the current study.

## Abbreviations

AAO: Indole‐3‐acetaldehyde oxidase
ACX: Acyl-coenzyme A oxidases
ALDH: Aldehyde dehydrogenase
AOS: Allene oxide synthase
CAD: Cinnamyl alcohol dehydrogenase
CAT: Catalase
FHB: Fusarium head blight
IAA: Indole-3-acetic acid
IAAld: Indole-3-acetaldehyde
IAM: Indole-3-acetamide
IAN: Indole-3-acetonitrile
IAOx: Indole-3-acetaldoxime
JA: Jasmonic acid
LOX: Lipoxygenase
L-TRP: L-Tryptophan
MeJA: Methyl jasmonate
N-OH-TAM: N-hydroxyl tryptamine
QTL: Quantitative trait locus
ROS: Reactive oxygen species
SA: Salicylic acid
TAM: Tryptamine
TDC: L-TRP decarboxylase
